# Do sentiments of professors feedback change after migrating from in-person to online modalities? Pre- and during COVID-19 experience

**DOI:** 10.1007/s10209-022-00943-2

**Published:** 2022-11-13

**Authors:** Lilia Carolina Rodríguez-Galván, Asad Abbas, Anil Yasin Ar, Beatriz Garza-González, Patricia Esther Alonso-Galicia

**Affiliations:** 1 School of Business, Tecnológico de Monterrey, Querétaro, Mexico; 2 Faculty of Informatics, Universidad Autónoma de Querétaro, Querétaro, Mexico; 3 Writing Lab, Institute for the Future of Education, Tecnológico de Monterrey, Monterrey, NL 64849 Mexico; 4 School of Government and Public Transformation, Tecnológico de Monterrey, San Pedro Garza García, NL Mexico; 5 School of Business, Department of International Business and Logistics, Tecnológico de Monterrey, Querétaro, Mexico; 6 Faculty of Languages and Literature, Universidad Autónoma de Querétaro, Querétaro, Mexico; 7 Institute for the Future of Education, Tecnológico de Monterrey, Querétaro, Mexico

**Keywords:** COVID-19, Educational innovation, Higher education, Sentiment analysis, Professors, Feedback

## Abstract

The COVID-19 pandemic forced higher education institutions to alter how they offer classes at an unprecedented pace. Due to ambiguities and lockdown restrictions, the transition phase negatively impacted students’ and professors emotions. As a result, lecturers had to cope with unfamiliar online class teaching responsibilities and develop new teaching dynamics. This work aims to analyze one of the most adversely affected procedures of teaching, the written feedback provided to students. This research strives to explore whether the professors’ feedback style altered from face-to-face education to online education on digital platforms during the COVID-19 restrictions. This exploratory-design study uses a mixed methodology to explain the subject on hand based on data collected from 117 undergraduate students. Sentiment lexicographers are utilized to address and identify the emotions expressed in the texts. Trust was the most frequent emotion expressed in face-to-face and online courses. It is also observed that the sentiments of joy and sadness changed significantly among online and face-to-face groups based on the professors’ feedback style and approach. Finally, the study reveals that the joy words and the sadness words associated with the learning process are the most commonly utilized sentiments. This study suggests that when the courses transitioned from face-to-face to online learning, the professors’ feedback changed to a more positive feeling that expressed appreciation for the students’ work, encouraging them to strive for their complete academic development, and usher them into a better learning environment.

## Introduction

The COVID-19 pandemic restricted social mobility, reduced social interactions, and introduced physical distancing. The crisis provoked sudden changes, uncertainty, and stress, disrupting the routines of everyday life [1]. It is estimated that 138 countries closed schools worldwide, and other countries implemented regional or local closures [2]. In higher education, a global survey by the International Association of Universities (IAU) reported that the shift from face-to-face to distance teaching came with challenges in infrastructure, competencies, and pedagogies for distance learning [3]. The official report for the government of Mexico reveals that in higher education during the school period (2019–2020), approximately ninety thousand people did not finish formal education; 44.6 percent of the dropouts were related to COVID-19 causes [4].

Due to the pandemic, professors and students were negatively affected emotionally by the lockdowns [5]. With increasing pressure to adapt to new realities, their experienced anxiety levels are increased. Ambiguous teaching and learning environments further fuel the fear of not performing their responsibilities using unfamiliar technology [6]. The sudden quarantines created additional uncertainty as professors and students had to adjust their conduct and incorporate new elements [7]. Most scholars attributed the uncertainty to the change in the mode of education, not considering whether the relationships among students and professors were positively or negatively impacted by changes in their interactions through media platforms.

The relationships between pupils and educators can be explored through the “emotions” perspective. The student may feel more secure and motivated with the professor’s support. In this regard, feedback, which enables professors to adjust their teaching-learning process according to the needs of each student, requires applying cognitive and affective elements to convey relevant messages to students that result in desirable learning outcomes [8, 9]. Hence, students and professors must communicate in a coherent, straightforward, and emotionally balanced manner. Failing to provide balanced feedback may strike fear among students, which may lead students to lose their confidence in coursework, especially when using digital communication platforms [10]. On the other hand, over-the-top positive feedback could be detrimental to the success of the students since it would create an unrealistic entitlement to success in a virtual learning environment. Thus, it is a chief concern of STEM-related education literature to focus on how emotional fluctuations during the COVID-19 period impacted interaction among students and professors.

Another perspective to be considered is the role of socialization in education. Education is enriched by the interactions among students, their peers, and lecturers. These accompany the formative process and the non-verbal expressions in language, which stimulate the learning process emotionally and affectively [11]. In online education, spontaneous, out-of-classroom contacts and interactions are few or completely absent [12]. During the online schooling period of the quarantine, students lacked face-to-face contact, which affected the learning process. Handling environmental uncertainty and emotional challenges has been burdensome leading to high dropout rates [13, 14].

During the learning process, feedback is an opportunity to provide information that keeps individuals on track with their goals. Formative feedback strengthens the students’ future performance [15]; affective feedback can promote students’ deep and relevant learning [16]. Based on the mentioned notion, the following research question is formulated: Did the professors change the affective component of their feedback during the change from face-to-face to online courses caused by the quarantine? The primary objective of this research was to determine whether the professors’ feedback changed and to understand how the affective components changed during the transition from face-to-face to online education caused by the COVID-19 quarantines. To achieve this objective, we conducted an empirical study analyzing professors’ feedback in one observation group. We employed a mixed methodology using coding schemes and a natural language processing algorithm for sentiment analysis in text analyses.

## Literature review

Notable philosophers and psychologists have developed concepts and definitions of emotions. They have established a relationship between emotions and beliefs, resulting in physiological reactions depending on the circumstances in which they occur and the originating behaviors. They have also documented the relationship between emotions and the cultures of social groups. Studying emotions requires complex and relevant investigation. Thus, it is essential to clarify the meaning of these two concepts, i.e., emotions and sentiments. In psychology, scholars illustrate that emotions intrinsically manifest themselves as conscious or unconscious reactions. According to Dewey [17], emotion has three components: an idea, a sentiment, and a behavior. Moreover, when emotion passes through consciousness, a reflective process gives rise to sentiment. Thus, sentiment is a conscious recognition of the perceived emotion. Therefore, sentiments are the emotions that have been identified and verbalized. The verbalization of emotion is related to a conscious cognitive process that reflects the individual’s emotional vocabulary [18].

**Fig. 1:**
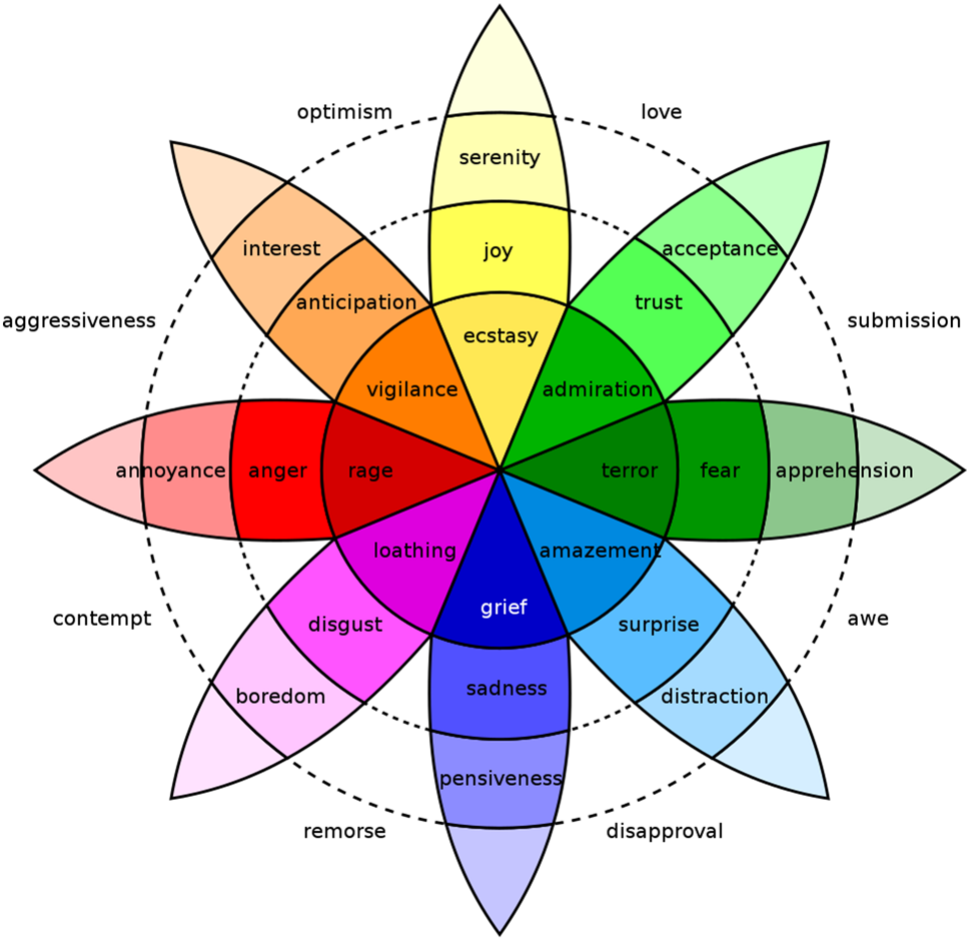
The Wheel of Emotions by Robert Plutchik [19]

Plutchik [19] formulated the Psychoevolutionary Theory in his study of emotions. His work has made valuable contributions to understanding emotions and emotional balance for survival. His theory has been used in education [20] with expressions [21] of emotions linked to artificial intelligence [22] in robotics [23]. Plutchik’s theory is visually represented in the so-called “Wheel of Emotions” (see Fig. 1).

This visual representation incorporates relevant elements of his theory, graphically identifying eight primary emotions, their relationship, and the new and opposing emotions brought about by them. Below is a brief explanation of each.

First, note that eight different colors represent the primary emotions of joy, confidence, fear, surprise, sadness, aversion, anger, and anticipation. Each is displayed with a color that changes in hue. These tone changes represent the relationships among different emotions according to their intensity. In the center of the figure are the deepest shades of color, representing emotions of the highest intensity. Progressing outward are the more subdued colors and emotions. For example, trust lies between admiration and acceptance and is illustrated by its color intensity.

The visual representation is organized so that emotions and their opposites are illustrated. For example, the opposite of “joy” is illustrated as “sadness”; the opposite of “anticipation” is illustrated as “surprise”; the opposite of “fear” is illustrated as “anger”; the opposite of “disgust” is illustrated as “trust”. Note in the graphic that two or more emotions can also exist simultaneously.

Plutchik [24] called the presence of two simultaneous emotions ”diades”. They are located on the plane between two colors in the wheel. For example, simultaneous trust and joy generate love; serenity and interest produce optimism. These spaces illustrate the possibility of an infinite combination of emotions and color hues in the wheel. The Wheel of Emotions was used in this research to guide the classification of the eight emotions to understand the feedback change.

## Methodology

The present study utilized a mixed methodology with content analysis (quantitative) and thematic analysis (qualitative). Both involved coding content precisely and carefully [25, 26]. The content analysis determined the presence of certain words and quantified them for analysis. The thematic analysis involved an emergent and interactive process of interpretation with some thematic structure for outcomes. Both approaches were viewed as complementary in the text analysis [27]. Content analysis is a quantitative technique, which requires human or computer coding. Diverse software programs are available to analyze texts, for example, R packages such as VADER, SentWordNet, BERT, and NRC. This study utilized the NRC sentiment analysis lexicon library in R programming. During coding, eight basic word classifications of Plutchik’s “wheel of emotion” were employed. Aligning with Borth et al. [28], the wheel of emotion was also utilized to enhance artificial intelligence algorithms involving images.

### Sample and procedure

The sample comprised undergraduate students from Tecnologico de Monterrey, a private university in Mexico. This selection was for convenience to have access to the information required. One hundred seventeen undergraduate students participated in the research: 52 students during the pre-COVID-19 period (named pre-group in this research) and 65 students during the COVID-19 period (named post-group in this research). The pre-group participated from August 2018 to December 2019, and the post-group from January 2021 to December 2021. The pre-group was taught using the face-to-face teaching model, and the post-group learned in the distance model. The researchers eliminated personal data identifiers to safeguard personal privacy. The data set used a unique identifier for each student while maintaining personal data separately for control purposes [29].

The undergraduate students were in computer systems engineering programs and enrolled in courses from three different schools: Engineering and Sciences, Humanities and Educations, and Business. The written feedback was registered in the course platform developed for evaluation and feedback purposes in these courses (https://app.gladio.com.mx).

The participating professors taught disciplinary courses (project management and evaluation, software quality and testing, software engineering project management, software design and architecture) and transversal courses (business ethics, entrepreneurial, and communication). Five professors were in the pre-group and three in the post-group. There were cases where one professor was responsible for two courses. To collect the feedback given to the undergraduate students during the evaluation, we extracted the written feedback comments from the online platform used in the courses. During a course, the professors provided comments after one-on-one interviews and evaluated the students’ evidence [30]. The pre-group interviews were conducted face-to-face and the post-group through a Zoom platform.

### Data analysis

For the content analysis, the text was structured in an XML file and translated from Spanish to English to be analyzed with a sentiment analysis algorithm. The English lexicon dictionary has more words registered than the Spanish. The text was pre-processed and cleaned, removing stop words, unnecessary punctuation, words in uppercase, misspellings, and we looked for incorrectly used exclamation marks or question marks. We used R Studio with a sentiment analysis algorithm with the Syuzhet library. Once the words were extracted and classified, we used Jamovi software to run descriptive analysis and independent samples t-test comparisons, observing significant differences among the groups (pre- and post) when exploring sentiment changes.

For the thematic analysis, we read the notes and looked for keywords, trends, themes, or ideas that would help outline the emotional tone of the words. For coding, we adapted the guide offered by Hennessy et al. [31, 32] for our research purpose. To understand the sentiment evoked by feedback given to the students, we began with content analysis. Subsequently, we conducted a thematic analysis with the specific sentiments that changed in pre- and post-analysis. We noticed that this approach provided a deeper understanding of the complexities of the changes in affective dimensions in feedback during the shift from the face-to-face to distance learning modalities brought about by COVID-19.

## Results and discussion

In this study, the mixed methodology approach was leveraged to address content and text sentiments effectively. While the t-test ensures a sound comparison between tested pre- and post-COVID-19 groups, the sentiment analysis revealed the thematic classifications and sentimental boundaries. As a result, the depth of the study reaches beyond exploring solely the word patterns or what a stand-alone empirical study could reveal. Moreover, it reveals the significant difference while illustrating the patterns of meanings, context, and communication approaches. Therefore, a similar methodical approach can be replicated in inter-disciplinary research that addresses communication, social media, and marketing contexts. The following section provides the integration of the content and thematic analysis.

### Descriptive statistics

For this empirical exploratory study, we obtained 875 sentences from the professors’ written feedback in the online course platform, 465 sentences for the pre-group and 410 sentences for the post-group (see Table 1). Exploratory studies are generally based on non-probabilistic samples, and primary data is analyzed for the study’s context and purpose [33]. In this case, the sentences analyzed considered different types of students in the learning process to obtain more variety.

**Table 1:** Pre- and Post-Group description

Academic period	Number of students	Number of sentences
Pre-Group (2018-2019)	52	465
Post-Group (2021)	65	410

Of the 117 students, 85 percent (99) were male, and 15 percent (18) were female. This composition was the same in the pre- and post-group (see Table 2).

**Table 2:** Pre- and Post-Group composition

	Number of students		
Academic period	Total	Male$^{1}$	Female$^{2}$
Pre-Group (2018-2019)	52	44	8
Post-Group (2021)	65	55	10
Total	117	99	18

$^{1}$ 85 percent from the total

$^{2}$15 percent from the total

### Changes in positive–negative sentiments in professors’ feedback

We applied the sentiment algorithm to obtain the number of words classified as positive or negative sentiments in these sentences during the content analysis. Overall analysis shows a positive pattern in both groups (see Table 3). From 2000 words in the pre-group, 86 percent were classified as positive, and from 1,906 words in post group, 89 percent were positive. We found more positive percentage words in the post-group.

**Table 3:** The number of words classified by positive/negative tone

	Pre-Group (2018-2019)		Post-Group (2021)	
	Number of words	Percent	Number of words	Percent
Positive	1717	86	1696	89
Negative	283	14	210	11
Total	2000		1906	

**Fig. 2:**
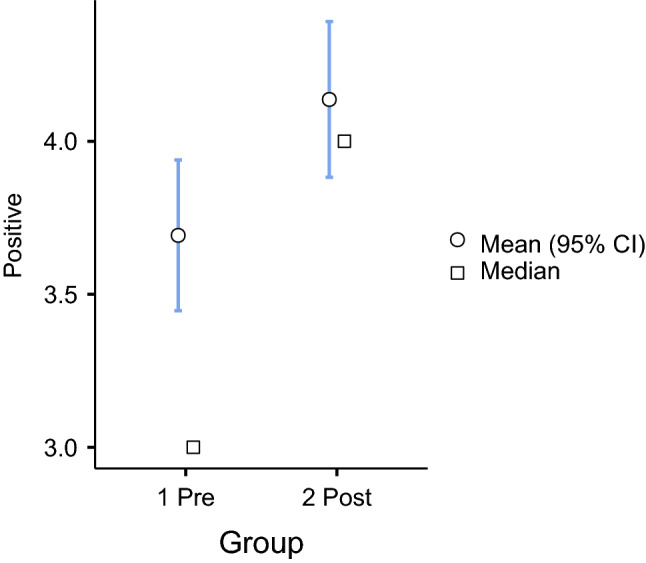
Independent samples t-test among pre- and post-group for positive sentiment

**Fig. 3:**
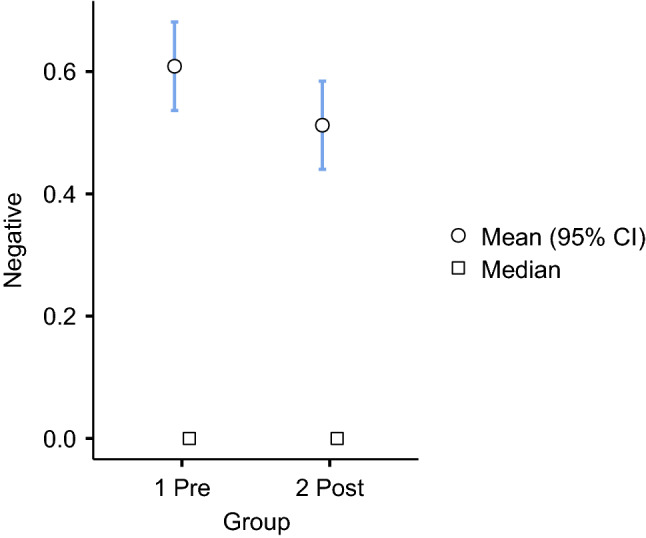
Independent samples t-test among pre- and post-group for negative sentiment

To understand what happened at the sentence level, we applied an independent sample t-test to measure statistical differences among the groups (see Figs. 2 and 3).

It can be observed that, on average, each sentence of professors’ feedback was more oriented to positive words in the post-group than in the pre-group. We observed, on average, 3.6 positive words in the pre-group and 4.1 in the post-group (with a 95 percent confidence interval). Regarding negative words, on average, 0.6 negative words in the pre-group and 0.5 for the post-group (with a 95 percent confidence interval) could be observed. The differences among the groups favored positive words, and these differences were significant at less than.05 (see Table 4).

**Table 4:** Independent samples t-test significance differences among pre- and post-group

Sentiment	Statistic	df$^{3}$	*p*
Negative	− 1.842	873	0.066
Positive	2.454	873	0.014 $^{4}$

$^{3}$ df$=$degrees of freedom

$^{4}$ Level of significance p $<.05$

The results suggest that professors adjusted their emotional feedback words for teaching-learning purposes. Professors and undergraduate students were affected by the COVID-19 context and emotions. It is noticed that the professors maintained a positive tone in their words and even increased it in the post-group. We suggest that this was a practice they consciously or unconsciously adjusted to mitigate the classes’ experience in the context of uncertainty. Frey et al. [34] argue that all professors incorporate affective elements into their teaching practice even if they are unaware. Villavicencio and Bernardo [35] established that one of the main steps to incorporating the affective dimension in teaching practices is recognizing and identifying its presence.

### Changes in primary sentiments in feedback

According to Plutchik [24], whose Emotion Theory work has made valuable contributions to understanding emotions [20, 22, 23], there are eight primary sentiments and a possibility of an infinite combination of them. The primary sentiments are joy, trust, fear, surprise, sadness, disgust, anger, and anticipation. The algorithm we used in this research classifies the words into these eight primary sentiments. Based on sentiment analysis, we observed words inclined to invoke trust, anticipation, and joy. However, we noticed changes in the pre- and post-group, mainly in the joy and sadness sentiments (see Table 5 ).

**Table 5:** List of number of words classified by sentiment

Sentiment	Pre-Group (2018-2019)		Post-Group (2020-2021)	
	Number	Percent	Number	Percent
Trust	1160	41	1145	41
Anticipation	665	24	615	22
Joy	405	14	530	19
Surprise	212	8	194	7
Fear	170	6	121	4
Anger	81	3	81	3
Sadness	99	4	54	2
Disgust	24	1	22	1
Total	2816		2762	

**Fig. 4:**
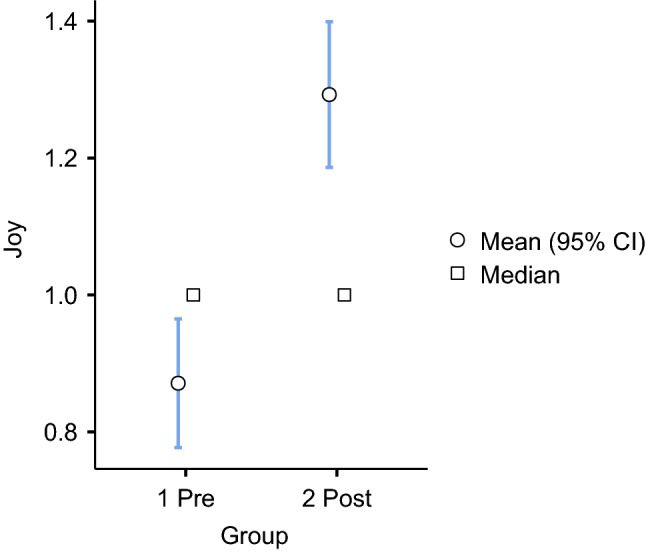
Independent samples t-test among pre- and post-group for joy

**Fig. 5:**
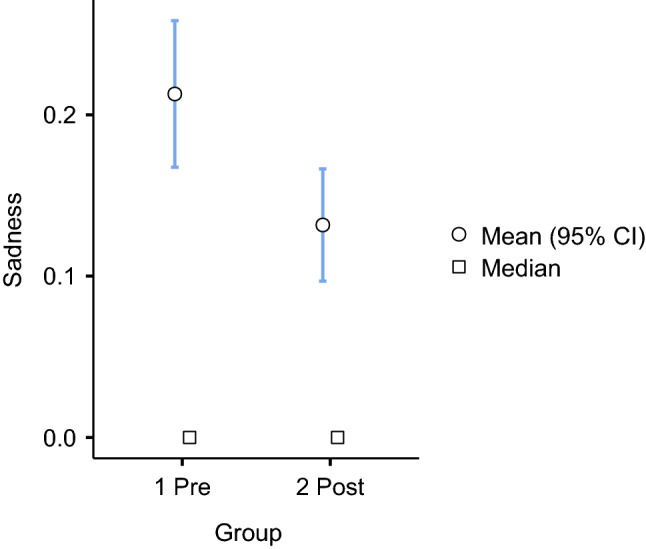
Independent samples t-test among pre- and post-group for sadness

Of 2,816 words in the pre-group, 41 percent (1,160 / 2,816) were related to trust and the same percentage in the post-group (1,145 / 2,762). The studies of sentiments in education have shown that trust facilitates class participation and establishes support relationships [36]. This feeling also helps students learn from their group mistakes. Trust builds an atmosphere that provides opportunities to learn from mistakes, communicate better, and construct a better learning environment [37]. Among the eight primary sentiments, we observed an increase in joy words from 14 percent to 19 percent in the post-group. Significantly, words related to sadness decreased from 4 percent to 2 percent. These results suggest that the professors’ feedback became more joyous and conveyed less sadness when the courses transitioned from face-to-face to online learning modalities in the COVID-19 context. To have greater confidence, we performed an independent sample t-test analysis to check if there were statistically significant differences among the groups in the joy and sadness sentiments at the sentence level (see Figs. 4 and 5).

Figures 4-5 show significant differences in words related to joy and sadness. The average words per sentence related to joy increased from 0.85 in the pre-group to 1.29 in the post-group. Words related to sadness decreased from 0.20 in the pre-group to 0.13 in the post-group. A significant difference was found in both sentiments with a 95 percent confidence interval (see Table 6).

**Table 6:** Significant differences in each sentiment among Pre- and Post-groups

Sentiment	Statistic	df $^{5}$	p
Trust	2.363	873	0.018
Anticipation	0.716	873	0.474
Joy	5.842	873	<.001 $^{7}$
Surprise	0.373	873	0.709
Fear	− 1.742	873	0.082
Anger	0.747	873	0.455
Sadness	− 2.726	873	0.007 $^{6}$
Disgust	0.129	873	0.897

$^{5}$ df = degree of freedom

$^{6}$ Level of significance p <.01 $^{7}$ Level of significance p <.001

Joy sentiments were more frequent in the feedback sentences to undergraduate students during the COVID-19 period (post-group). The words associated with sadness were less prevalent. To better understand the joy-sadness change in the feedback, we conducted a thematic analysis, explained in the next section.

### Joy-Sadness sentiments in feedback

We analyzed the sentences associated with joy and sadness to identify the use of emotion in the learning process. Coding identified the incidence of critical words and some corresponding words that could appear together in repeated cases [32]. The coding guide created for analyzing classroom dialogue helped us analyze dialogic feedback during the evaluation process [31]. We noticed that joy was more related to words associated with achieving goals in the students’ work. We observed that some words frequently appeared together, like ”applause”, ”performing”, ”pretty”, and ”verification”. We noticed that this category was also related to the elaboration of the work. Appreciation is an element in the feedback process, and these results suggest that appreciation elements are associated with joy sentiments. Neuroscience has presented the relevance of joy in education to teaching practices that promote learning [38]. The feeling of joy garners interest in being repeated to maintain stable humor and overcome sadness. For learning purposes, joy is associated with achievement and the triumph of attaining the results [39]. We also observed that sadness was more related to words associated with invitations to reflect. The words that appear together in sentences related to sadness emotions were reasoning, willingness, moving, handles, planning, attempts, and conscience. This category was related to reflection on the learning process. During the feedback process, the professors must give orientation and guidance on achieved goals while pointing out the failures as well as poor performances. However, this process could lead to negative emotions being salient among undergraduate students. We observed that fewer words were associated with sadness in a sentence when the sentence also invoked other sentiments like trust and joy. This observation pertained mainly to the post-group, where the sentences had different words associated with different emotions. To understand these mixed-sentiment words in a sentence, we analyzed the transcriptions of some sentences. Notably, in the studies of negative emotions like sadness, it has been observed that these could be construed as beneficial under some circumstances [40–42]. Thematic analysis is an inductive qualitative analysis and works with transcriptions. It is essential to understand that the transcription does not represent reality because communication is complex and involves many influential verbal and non-verbal communication elements [43]. Our results showed that the words expressing sadness decreased when the joy emotions were emphasized in a sentence. Some examples of feedback transcriptions illustrating the joy and sadness emotions are provided in the following:

”She has been very involved in the team’s performance, implementing actions like working all at the same table, reactions like applause in meetings, and follow-up to presentations. To advance the level, I expect evidence of the outcome of the meetings and the support of the team.” (Pre-Group)

”All right, (name), it is noted that you have a willingness to listen and make decisions in an informed way, considering others. This moment of reflection where ethical reasoning resides is right where you have to pay attention. I agree with you about what this training is. I invite you to make a kind of log in which you write what you reflect on situations of conflict (personal or group) to eventually have it in future or current situations to build an ethical culture in the department. I recommend that you consciously fine-tune this response in this reflection, both the consequences and affected values in each of the actors.” (Pre-Group)

”All right. Continue to consolidate happy, high-performing teams.” (Post-Group)

”It documents your experience in developing your competence very well. There are some verbal quotes and bodily reactions you can fine-tune. Do not lose the reflection on your documentation and rather integrate all the feedback you receive into your practices.” (Post-Group)

Thus, during the transition from a face-to-face course to an online course, the professors incorporated and adjusted their feedback with affective elements. We noticed more words of joy and fewer words of sadness in the written feedback given to undergraduate students.

## Conclusion

Coming back to the research question and on the basis of our empirical results and discussion, we can conclude that professors’ feedback changes its emotional tone in the transition from a face-to-face to an online course due to COVID-19. Moreover, the outcome of this research suggests that professors adjust, consciously or unconsciously, this affective component to reduce stress and uncertainty and offer more joy words. The main theoretical contribution of this research is knowledge about feedback and its affective components to understand and use their potential for adaptive changes in uncertain circumstances such as COVID-19. We analyzed the changes in certain emotions (joy, sadness) in written feedback and identified how it was used for pedagogical purposes. This work is valuable because educational research on the relationship between emotions and feedback is scarce [44].

The practical implication of this research is a better understanding of the affective dimension in feedback. Professors can use this study to understand their affective style of giving feedback and explore how they use emotions to guide students’ success. For example, in our study, the joy words were often related to goal achievement and appreciation for the students’ efforts and the results. Also, the research was valuable in understanding some mixes of emotions that could appear together in a sentence to point out some mistakes and create an appropriate condition to appreciate future corrections. We observed that sadness could arise when professors invited reflections on the learning process.

Secondly, educational actors can understand the relevance of training professors in emotional intelligence for pedagogical purposes. Exploiting the findings of this research could initiate innovative actions with pedagogical design approaches that use technology to mediate affective components in feedback processes in challenging, uncertain learning contexts. Also, technological advances in analytics learning allow the possibility of exploring the impact of emotions through different formats and understanding them.

## Limitations and future directions

One limitation of the present research was the chosen context, because of the COVID-19 quarantine: the students’ and professors’ environment was, and still has been, uncertain. Since it is a new phenomenon currently evolving, a follow-up study may shed more light on the discussed matter. New insights, pedagogical approaches, and longer spawn of students’ adjustment time may reveal a better understanding of the subject.

Another limitation is related to the nature and scope of the project. This study is funded by the Tecnologico de Monterrey under the premise that the work that is completed would be investigating the learning experience of the Tecnologico de Monterrey’s students, a private university in Mexico. Therefore, the sample available for the present study was limited. In addition, there are significant differences between public and private universities in terms of the availability of e-learning tools and the ability to use technology in teaching environments. Since digital tools are heavily invested in delivering top-notch education in Tecnologico de Monterrey and are seen as an important enabler of academic training, these results may not be the best representation or indications of the public university context. Thus, a future study that addresses public universities could lead to a better understanding of the bigger picture.

Academic stress, the lack of time management skills [45] and students’ inability to manage anxiety can lead to adverse effects in students’ learning [46]. Therefore, future research could focus on extending the understanding of how to employ feedback in various teaching modalities to regulate emotions in regard to stress or anxiety in order to achieve a desirable learning outcome.

Furthermore, it is necessary to consider other types of courses or other models to explore subject on hand. Sentiment analysis has been used to visualize emotions in opinion studies from the students’ perspective [47–50] and the parents’ perspective [51]. However, it is vital to extend the research into the role of emotions in professors’ feedback using sentiment analysis. Extended research could lead to understanding the sentiments during feedback using other technologies, such as bots [52] or different formats like audio or video. Other researchers could explore the use of other specific sentiments and their relations (such as joy-sadness) assertively in feedback to encourage the students in the learning process. To further extend this research, it might be beneficial to understand how to use technology [53] to manage emotions in feedback to point out a failure or poor performance while simultaneously encouraging students to maintain interest and motivation in their learning.

## Data Availability

The dataset is available from the first author (Lilia Carolina Rodríguez-Galván) on reasonable request. Conflicts of Interest: No conflict of interest to report.
